# Effect of Rat Medicated Serum Containing You Gui Wan on Mouse Oocyte *In Vitro* Maturation and Subsequent Fertilization Competence

**DOI:** 10.1155/2014/152010

**Published:** 2014-10-28

**Authors:** Xiao-Hui Jiang, Yan-li Deng, Hua Lu, Heng Duan, Xia Zhen, Xiang Hu, Xin Liang, Shang-mian Yie

**Affiliations:** ^1^Department of Gynecology and Obstetrics, The Second Medical College/Teaching Hospital, Chengdu University of Traditional Chinese Medicine, No. 15, Fourth Segment, Remin Nanlu Road, Chengdu, Sichuan 610041, China; ^2^College of Traditional Chinese Medicine, Chongqing Medical University, 1 Yixueyuan Road, Yuzhong District, Chongqing 400016, China

## Abstract

You Gui Wan (YGW) is a classic herbal formula in traditional Chinese medicine (TCM) used for the clinical treatment of infertility. This study was to explore whether YGW has an impact on mouse oocyte maturation *in vitro* and subsequent fertilization competence. Rat medicated serum containing YGW was prepared by orally administrating YGW. Mouse immature oocytes were cultured with YGW medicated serum and compared to those cultured with or without normal rat serum or follicle-stimulating hormone (FSH). YGW medicated serum significantly increased the percentages of matured oocytes when compared to the groups with or without normal rat serum (*P* < 0.01). Furthermore, YGW medicated serum increased the rate of *in vitro* fertilization (IVF) when compared to the groups treated with FSH and with or without normal rat serum (*P* < 0.001). YGW medicated serum also had significant effects on the mRNA expressions of PKA, CREB, MAPK, PKC, PKG, and MPF and the concentrations of cAMP, cGMP, and NO in matured oocytes. These results indicate that YGW can promote mouse oocyte maturation and IVF *in vitro*. Signaling pathways, such as the cAMP/PKA/MAPK, the PKC-MAPK, and the NO-cGMP-PKG pathway, which are similar to those induced by FSH, may be responsible for this action.

## 1. Introduction

Traditional Chinese medicine or TCM has a long history of treating infertility [1]. According to TCM theories, which are completely different from Western medicinal theories, the “kidney” has the ability to store congenital essence from parents, as well as the ability to control reproduction, growth, menstruation, conception, and aging in women [2]. Moreover, TCM views disease as a result of disharmony or imbalance between two opposite and yet complementary forces: Yin and Yang [2]. As such, a “Kidney Yin” or “Kidney Yang” deficiency is at the root of many reproductive disorders in both males and females. For example, published surveys have shown that IVF patients predominately fall into “kidney” deficiency patterns, with “Kidney Yang” deficiency being the most prevalent followed closely by “Kidney Yin” deficiency [3]. Thus, a major purpose of TCM therapies is to reinforce the “Kidney Yin” or “Kidney Yang” deficiency in patients.

Typically, individual Chinese herbs are not considered very useful in treating infertility. Therefore, in the majority of cases, herbal formulas are prescribed which may consist of a dozen or more herbs. Herbal formulas can be administered as pills, powders, tablets, or decoctions (teas), with each method of administration being equally effective. In an earlier clinical study, we used a TCM formula called Yangjing Decoction (YJD) to treat 36 women with infertility and observed that YJD could promote follicular development and blood supply in the ovary and uterus [4]. The YJD formula was also found to stimulate the ovulation of 26 infertile patients in another clinical study [5]. Moreover, in a recent animal study, we found that another TCM formula called zircon granules (ZG) can promote follicle development in immature rats after rectal administration [6].

You Gui Wan (YGW) is a classic notification formula in TCM used for the clinical treatment of infertility that reinforces the “Kidney Yang” deficiency. The main components of the YGW formula are similar to those found in the YJD and ZG formulas, such as* Rhizome Chuanxiong, Radix Rehmanniae Preparata, Barbary*,* Cuscuta chinensis Lam.,* or* Fructus Corni*, suggesting that these formulas may have similar pharmaceutical functions in promoting oocyte development and maturation.

Based on these clinical and pharmaceutical observations, we hypothesized that YGW could promote oocytes maturation. To verify our hypothesis, we designed our present study to examine whether or not YGW has any impact on oocytes maturation by using assays of oocyte* in vitro* maturation (IVM) and* in vitro* fertilization (IVF) following IVM. Further, it has been reported that serum pharmacology is more scientific and more befitting than the use of traditional pharmacology in TCM research [7, 8]. In traditional pharmacology, crude drugs are directly added into a culture system of cells or organs* in vitro*. By contrast, in serum pharmacology, drugs or drug compounds are first administered to animals orally. After a definitive period of time, the blood of the animals is collected to separate the serum. The result is a medicated serum which has undergone a series of biotransformation after digestion and absorption in gastrointestinal tract. The medicated serum is then added into a culture system of cells or organs* in vitro*. This approach is more similar to the environment in which drugs work* in vivo* [9].

Thus, in our study, we cultured immature mouse oocytes in the presence of rat medicated serum containing YGW. The percentage of matured oocytes and the rate of fertilization for the maturated oocytes were compared to controls, which included oocytes that were cultured with and without normal rat serum or FSH. In addition, we investigated the effects of the rat medicated serum on signal pathways that control oocyte maturation, including gene expressions of protein kinase A (PKA), cAMP-response element binding protein (CREB), mitogen-activated protein kinases (MAPK), protein kinase C (PKC), protein kinase G (PKG), and maturation-promoting factor (MPF), as well as concentrations of cyclic adenosine monophosphate (cAMP), cyclic guanosine monophosphate (cGMP), and nitric oxide (NO) in oocytes, in order to explore its possible underlying mechanisms.

## 2. Materials and Methods

### 2.1. Preparation of Herbal Decoctions and Rat Medicated Serum

YGW was obtained from Beijing Tong Ren Tang with a herb medicine license Number Z23020593 (Tong Ren Tang Co. Ltd., Beijing, China). YGW is composed of nine different herbal ingredients [9], including* Radix Rehmanniae Preparata*,* Fructus Corni Officinalis*,* Fructus Lycii*,* Colla Cornus Cervi*,* Cuscuta chinensis Lam.*,* Eucommia ulmoides*, *Radix Angelicae Sinensis*,* Cinnamomum, and Radix Aconite Lateralis Preparata*. Decoctions of YGW (0.5 gram/mL) were prepared by using standard methods [2], diluted with saline (preparation: saline, 1 : 1 v/v), and stored at 4°C prior to use.

The rat medicated serum was prepared according to the published protocols [10, 11]. Briefly, 28 Sprague Dawley rats, aged between 6 and 8 weeks old and weighing between 220 and 250 grams, were divided into experimental (*N* = 14) and control (*N* = 14) groups. The animals were supplied by the Laboratory Animal Service Center at the Sichuan Academy of Medical Sciences and maintained in an air-conditioned room with controlled a temperature of 22 ± 2°C, a humidity level of 45% to 65%, and a 12/12 hrs light/dark cycle. The animals were fasted for 16 hours, and then individually administrated with YGW decoction of 20 mL/kg body weight (equal to 5.0 gram YGW/kg body weight based on clinical dosage). The control group received intragastric injections of physiological saline. After 60 min of administration, blood was collected from retinal venous plexus and centrifuged. The collected blood was aliquoted into 10 mL ampoules and preserved at −80°C for future use.

### 2.2. Plasma Samples Preparation for HPLC Analysis

The rat medicated serum was obtained after the blood was centrifuged at 3500 rpm for 15 min at room temperature. One milliliter of the rat medicated serum was mixed with 5 mL of methanol and ultrasonicated for 20 min. The mixture was then centrifuged at 3000 rpm for 10 min at room temperature, and the obtained supernatant was evaporated to dryness* in vacuo*. The dried residue was reconstituted in 500 *μ*L of methanol and filtrated through a 0.45 *μ*m membrane filter (Millipore Co. Ltd., Tokyo, Japan), and an aliquot (20 *μ*L) was applied to the HPLC analysis.

The HPLC analysis was performed with a Shimadzu LC2010C HPLC gradient system. The HPLC conditions were as follows: column: Inertsil ODS-2C18 (4.6 × 250 mm, GL Science, Tokyo, Japan); column temperature: 30°C; mobile phases: linear gradient system of methanol (A) and 0.1% H_3_PO_4_ (B) where A/B: 10/90 (0 min), 70/30 (28 min), and 100/0 (38 min); flow rate: 1 mL/min; detection wavelength: 259 nm; and injection volume: 70 *μ*L.

### 2.3. Experimental Animals

The Animal Care and Use Committee at The Chengdu University of Chinese Traditional Medicine approved this study and its procedures as protocol #30472225.

One hundred and forty-eight female ICR mice, aged between 6 and 8 weeks old and weighing between 22 and 26 grams, and 10 male ICR mice, aged between 8 and 10 weeks old and weighing between 30 and 35 grams, were supplied by the Laboratory Animal Service Center at the Sichuan Academy of Medical Sciences. The animals were maintained in an air-conditioned room with a controlled temperature of 22 to 25°C, a humidity level of 45% to 65%, and a 12 hr light/dark cycle.

### 2.4. Preparation of Gametes

Ovaries were dissected after 48 hours of a single subabdominal injection of 10 IU of pregnant mare serum gonadotropin (Folligon, Intervet, Lane Cove, Australia), and placed in a 2 mL handling medium (HEPES buffer with 10% human serum albumin). Antral follicles were pierced with sharp fine forceps to release immature cumulus-oocyte complexes (COC). Sperm was obtained from the cauda epididymides of male mice. The cauda epididymides were dissected out from the body and washed with PBS. The cauda epididymides were then transferred into a previously equilibrated (37°C at 5% CO_2_ in air) 2 mL medium (HTF with 10% serum protein substitute (SPS) under a thin layer of mineral oil). Sperm was passively released into the medium through a single slit in the base of the cauda epididymes.

### 2.5. Oocytes* In Vitro* Maturation Assay

Human tubal fluid (HTF) (Millipore, Billerica, MA, USA) was used as a basic culture medium for IVM. Retrieved immature COC were individually cultured in 20 *μ*L drops of the culture medium and randomly divided into four groups: blank control group (HTF only), serum control group (HTF with 10% normal rat serum), FSH (0.1 IU/mL, Laboratories Serono, Geneva, Switzerland) group (HTF with 100 U/L FSH), and rat medicated serum group (HTF with 10% rat medicated serum). The COC were first cultured in an atmosphere of 5% CO_2_ in air at 37°C under a thin layer of mineral oil (Chemicals Co., St. Louis, MO, USA) for 24 hours. The COC were then washed with serum-free medium and exposed to 100 IU/mL hyaluronidase (Type IV-S, Sigma) in the culture medium for 5 min where the cumulus cells were mechanically separated from the oocytes to obtain denuded oocytes. Maturation of the oocytes was observed under an inverted microscope (Olympus CK40) and a stereomicroscope (Olympus SZX9). Oocytes that had germinal vesicle breakdowns (GVBD) were considered to be at the start of meiosis, while oocytes that extruded the first polar body (PB1) were considered to be at nuclear maturation. All experiments were repeated at least three times on different occasions.

### 2.6. *In Vitro* Fertilization Assay

Oocytes that had undergone the first polar body extraction were first placed in sperm suspension for 3 hours. Next, the oocytes were washed and further cultured in 20 *μ*L drops of the HTF under oil at 37°C at 5% CO_2_ in air for 24 hours. Fertilization of the oocytes was observed under the inverted microscope. Oocytes, that had extruded a second polar body and two pronuclei that were visible 9 hours after the insemination, were considered to be fertilized. The cleavage rate of fertilized oocytes on day 3 of culture was recorded. All experiments were repeated at least three times on different occasions.

### 2.7. Measurement of the cAMP, cGMP, and NO Concentrations in Oocytes

To determine the concentrations of cAMP and cGMP in oocytes with nuclear maturation, different commercial ELISA kits (R&D System, Minneapolis, MN, USA, and Cayman Chemical Co., Ann Arbor, MI, USA) were used according to the manufacturers' protocols. The ELISA were evaluated at 450/630 nM. To assay the concentrations of NO in the oocytes, an NO kit (R&D Systems, Minneapolis, MN, USA) was used according to the manufacturer's protocol in which the enzymatic conversion product was evaluated at 540/690 nM. For a comparative analysis, each group had an equal number of oocytes in each assay and the results were presented as percentage changes with respect to the blank controls. All experiments were repeated at least three times on different occasions.

### 2.8. Real-Time RT-PCR Analysis

To assess the mRNA expressions of PKA, CREB, MAPK, PKC, PKG, and MPF, qPCR analysis was used as previously described [12]. Briefly, total RNA was extracted by using TRIzol reagents (Invitrogen, Carlsbad, CA, USA) and complementary DNA was prepared from the total RNA by using oligo primers and Moloney murine leukemia virus reverse transcriptase (Applied Biosystems Inc., Foster City, CA, USA). The qPCR analysis was performed with the SYBR Green mix (Applied Biosystems, Foster City, CA, USA) on an ABI Prism 7900 sequence detector (Invitrogen). Specific primers for PKA, CREB, MAPK, PKC, PKG, MPF, and *β*-actin are listed in Table 1. For each PCR product, the melting curve was determined by using the comparative threshold cycle number method [13], with the results being presented as percentage changes with respect to the blank controls. All experiments were repeated at least three times on different occasions.

### 2.9. Statistical Analysis

Statistical analysis was performed using the SPSS software package (Abacus Concepts Inc., Berkeley, CA, USA). All data were expressed as mean ± SEM and compared using the ANOVA and Duncan's test. *P* < 0.05 was considered to be statistically significant.

## 3. Results

### 3.1. Analysis of the Compounds Absorbed into the Blood after Oral Administration of YGW Decoction in SD Rats

**Figure 1:**
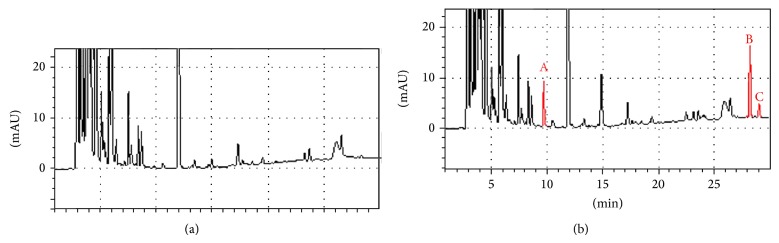
HPLC profiles of the rat medicated serum after the oral administration of the YGW decoction. (a) Normal rat serum; (b) rat medicated serum after 1 hour of the oral administration. Three distinct peaks (red color) are detected in the rat medicated serum, but not in the normal rat serum. This indicates that the rat medicated serum may contain active ingredients and/or metabolic components of the YGW decoction. NS: normal rat serum; MS: rat medicated serum.

Figure 1 shows the representative HPLC profiles for the normal rat serum and the rat medicated serum, respectively. Three distinct peaks can be detected in the rat medicated serum containing YGW when compared with the normal rat serum, which indicates that the rat medicated serum may contain active ingredients and/or metabolic components of the decoction.

### 3.2. Effect of the Rat Medicated Serum on Oocytes* In Vitro* Maturation

**Figure 2:**
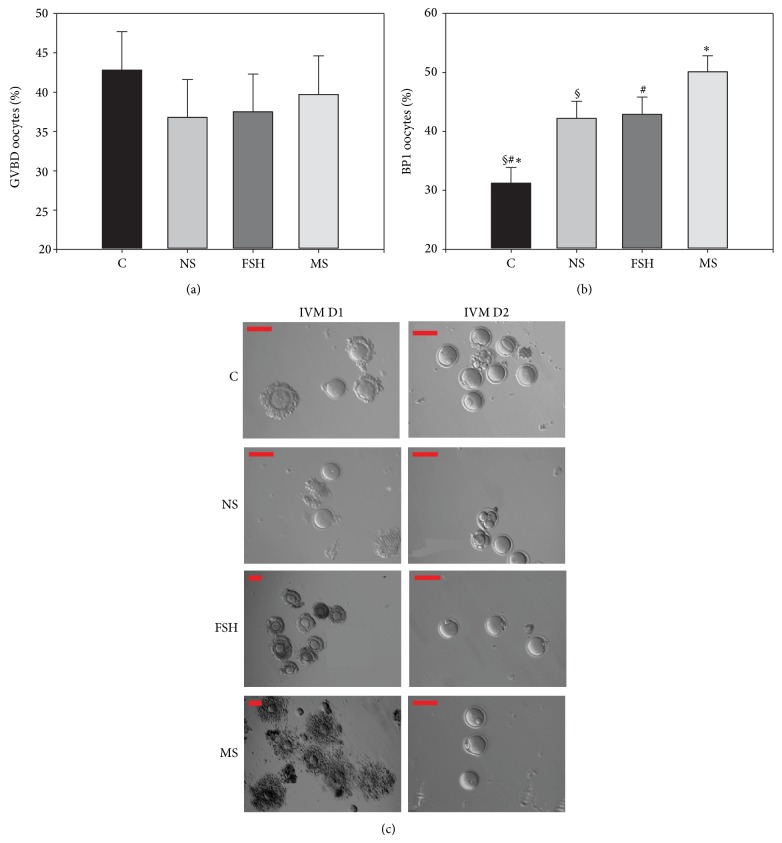
Effect of the rat medicated serum containing YGW on the percentage of GVDB oocytes and PB1 oocytes. (a) shows no significant difference in the percentage of GVBD oocytes observed among the groups on day 1 of the culture. (b) shows more oocytes with the PB1 stages found in the rat medicated serum, FSH, and normal rat serum groups when compared to the blank control group on day 2 of the culture (^*^ *P* = 0.0001 for rat medicated group versus blank control group; ^§^ *P* = 0.004 for FSH group versus blank control group; ^#^ *P* = 0.006 for normal rat serum group versus blank control group). (c) shows representative images of light micrographs of oocytes on day 1 and day 2 in the normal rat serum group, the FSH group, the rat medicated serum group, and the blank control group. Scale bar is set to 100 *μ*m. C, NS, FSH, and MS are abbreviations for blank control, normal rat serum, FSH, and rat medicated serum, respectively.

A total of 1179 immature oocytes were retrieved from the 148 female ICR mice. Of these, 292 were used in the blank control group, 277 were used in the serum control group (HTF with 10% normal rat serum), 275 were used in the FSH group, and 335 were used in the rat medicated serum group. After an* in vitro* culture of 24 hours, 42.8 ± 4.9% of the oocytes in the blank control group, 36.8 ± 4.8% of the oocytes in the normal rat serum group, 37.5 ± 4.8% of the oocytes in the FSH group, and 39.7 ± 4.9% of the oocytes in the rat medicated serum group underwent GVBD. As shown in Table 2 and Figure 2(a), no significant difference was observed among the groups, except in terms of the percentage of PB1 oocytes (31.2 ± 2.7% (blank control) versus 42.2 ± 2.9% (normal rat serum) versus 42.9 ± 2.9% (FSH) versus 50.1 ± 2.7% (rat medicated serum), *F* = 7.902, *P* = 0.0001, Table 2 and Figure 2(b)). Duncan's analysis further demonstrated that the percentage of PB1 oocytes in the rat medicated serum, FSH, and normal rat serum groups was significantly higher than in the blank control group.  Figure 2(c) shows representative samples of the effects of the rat medicated serum on oocytes* in vitro* maturation when compared to the other three groups.

### 3.3. Effect of Rat Medicated Serum on the Rate of IVF and Embryo Cleavage Rate

**Figure 3:**
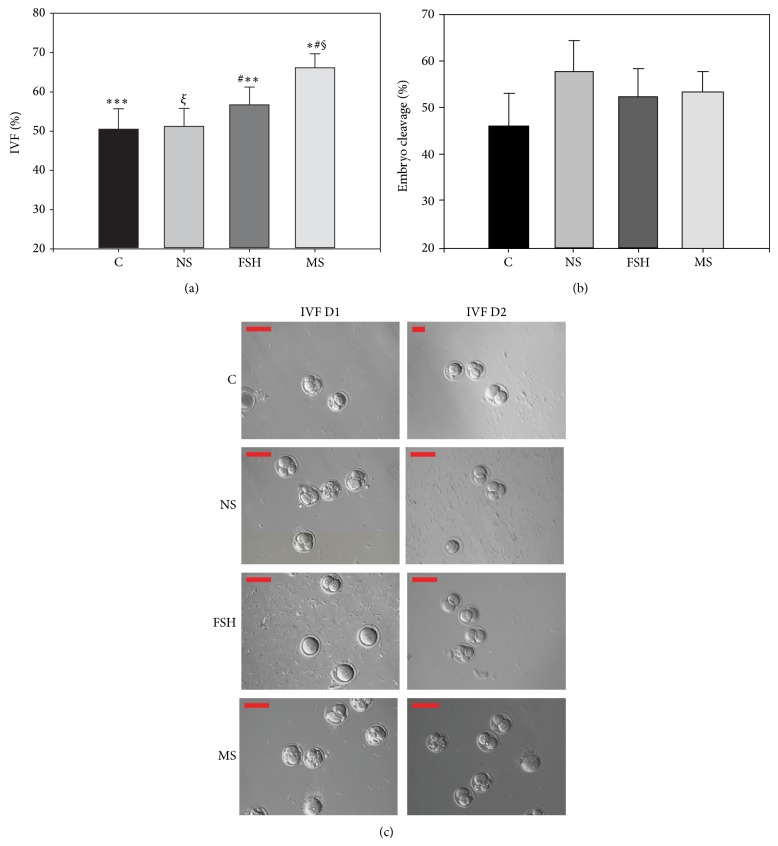
Effects of the rat medicated serum containing YGW on the percentage of* in vitro* fertilization and embryo cleavage. (a) shows a higher percentage of* in vitro* fertilization found in the rat medicated serum group when compared to the other three groups on day 1 after IVF (symbols ∗, §, and # indicate comparison of rat medicated group with blank control group, normal rat serum group, and FSH group, resp.; ∗∗ indicates comparison of FSH group versus blank control group). (b) shows no significant difference in the embryo cleavage rate found among the groups on day 2 after IVF. (c) shows representative images of light micrographs of the fertilized oocytes and embryos on day 1 and day 2 after IVF among the blank control group, the normal rat serum group, the FSH group, and the rat medicated serum group. Scale bar is set to 100 *μ*m.C, NS, FSH, and MS are abbreviations for blank control, normal rat serum, FSH, and rat medicated serum, respectively. IVM:* in vitro* maturation.

A total of 57.4% (289/494) of the PB1 oocytes were fertilized. Of these, 46 were in the blank control group, 60 were in the normal rat serum group, 67 were in the FSH group, and 111 were in the rat medicated serum group. As shown in Table 2 and Figure 3(a), the fertilization rate was significantly higher in the group treated with the rat medicated serum (66.1 ± 3.6%) than the other three groups (*F* = 25.08, *P* < 0.001). Duncan's analysis further demonstrated that the IVF rate in the rat medicated serum group was significantly higher than the IVF rate found in the blank control group (50.5 ± 5.2%, *P* = 0.004), the normal rat serum group (51.2 ± 4.6%, *P* = 0.0044), and the FSH group (56.7 ± 4.5%, *P* = 0.01). Further, the IVF rate in the FSH group was significantly higher than the IVF rate in the blank control group (*P* = 0.04), but not so when compared to the normal rat serum group. No significant difference in the IVF rate was found between the blank control and normal rat serum groups.

No significant difference in the embryo cleavage rate after fertilization was observed among the four groups (45.6 ± 7.2% (blank control) versus 57.8 ± 6.2% (normal rat serum) versus 52.2 ± 6.1% (FSH) versus 53.1 ± 4.7% (rat medicated serum), Table 2 and Figure 3(b)).  Figure 3(c) shows representative samples of the effects of the rat medicated serum on oocytes* in vitro* fertilization and embryonic cleavage when compared to the other three groups.

### 3.4. Effect of Rat Medicated Serum on cAMP, cGMP, and NO Concentrations in Oocytes

**Figure 4:**
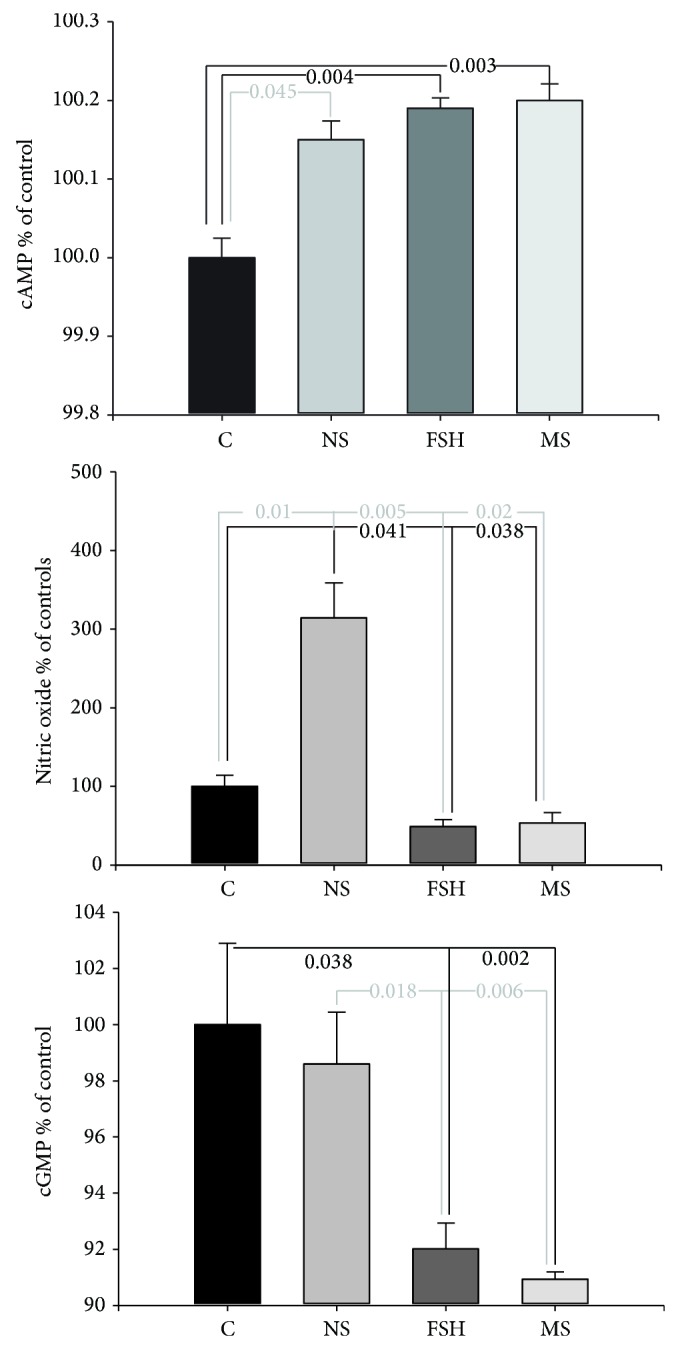
Effects of the rat medicated serum on concentrations of cAMP, cGMP, and NO in oocytes. The black line indicates comparisons between the blank control group and the FSH or rat medicated serum groups, while the grey line indicates comparisons between the normal rat serum group and the FSH or rat medicated serum groups. The numbers in the line breaks indicate *P* values calculated using Duncan's analysis. C, NS, FSH, and MS are abbreviations for blank control, normal rat serum, FSH, and rat medicated serum, respectively. IVF:* in vitro* fertilization.

As shown in Figure 4, significant difference in the concentrations of cAMP, cGMP, and NO in oocytes was observed among the groups (*F* = 10.81, *P* = 0.001 for cAMP; *F* = 7.02, *P* = 0.005 for cGMP; and *F* = 25.65, *P* = 0.0001 for NO). The Dunnett T3 analysis further demonstrated that the FSH group and the rat medicated serum group had higher concentrations of cAMP, but lower concentrations of cGMP and NO when compared to the blank control and normal rat serum groups.

### 3.5. Effect of Rat Medicated Serum on Gene Expressions in Oocytes

**Figure 5:**
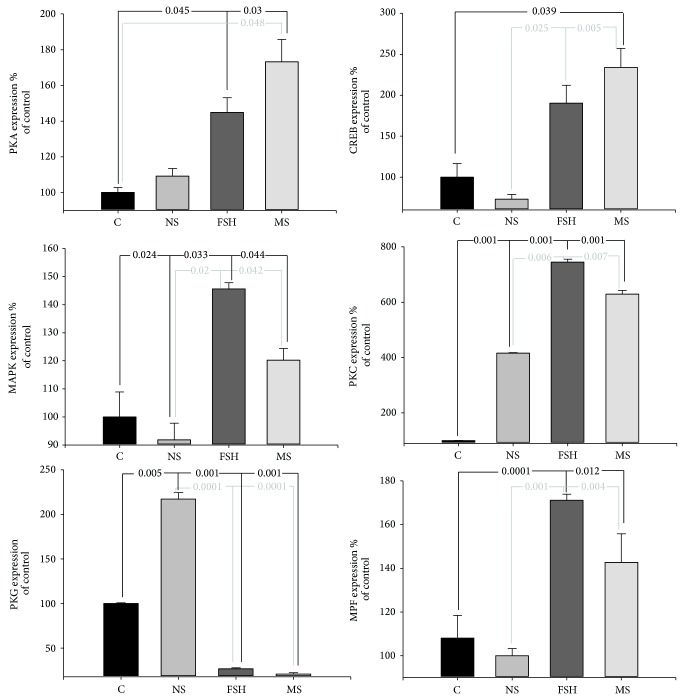
Effects of the rat medicated serum on the mRNA levels of PKA, CREB, MAPK, PKC, PKG, and MPF in oocytes. The black line indicates comparisons between the blank control group and the FSH or rat medicated serum groups, while the grey line indicates comparisons between the normal rat serum group and the FSH or rat medicated serum groups. The numbers in the line breaks indicate *P* values calculated using Duncan's analysis. C, NS, FSH, and MS are abbreviations for blank control, normal rat serum, FSH, and rat medicated serum, respectively.

Comparisons of the gene transcriptions PKA, CREB, MAPK, PKC, PKG, and MPF in oocytes among the groups are shown in Figure 5. The expressions of PKA, CREB, MAPK, PKC, PKG, and MPF were found to be statistically different among the groups (*F* = 4.43, *P* = 0.026 for PKA; *F* = 5.11, *P* = 0.019 for CREB; *F* = 3.38, *P* = 0.044 for MAPK; *F* = 7.21, *P* = 0.003 for MPF; *F* = 4.07, *P* = 0.03 for PKC; and *F* = 4.36, *P* = 0.025 for PKG). Further comparisons using the Dunnett T3 analysis showed that in the FSH and rat medicated serum groups, the expressions of PKA, CREB, MAPK, PKC, and MPF were significantly higher and the expression of PKG was significantly lower, when compared to the blank control or the normal rat serum groups.

## 4. Discussion

The process of oocyte maturation is very complex. In most mammals, oocytes are arrested at the diplotene stage, also called the germinal vesicle stage, of the first meiotic prophase until a surge of gonadotrophins from the pituitary stimulates the immature oocytes into resuming first meiosis and ovulation [14]. Morphologically, the resumption of meiosis is characterized by the disappearance of the nuclear membrane in the oocytes. The first meiotic division that progresses through metaphase I is manifested by the formation of the first polar body, with the oocytes being arrested again at metaphase II until fertilization [15]. The processes of follicular development, meiotic resumption, and subsequent ovulation in mammals are controlled by two pituitary derived glycoprotein gonadotrophins, FSH and LH [16].

According to TCM theory, the “kidney” houses the reproductive essence (jing) and governs reproduction. In modern terminology, the essence refers to eggs and sperm, the most primordial building blocks of reproduction [17]. While the “Kidney essence” correlates with eggs and sperm, the “Kidney Yin” and “Kidney Yang” can be thought of as the hormonal underpinnings of the reproductive system [17]. Because YGW is used in patterns with “Kidney Yang” deficiency, it may regulate the endocrine function in order to promote oocyte maturation and subsequent fertilization competence.

In our present study, by using IVM and IVF assays, we showed that rat medicated serum containing YGW can enhance oocyte nuclear maturation* in vitro* when compared to the blank control and normal rat serum groups. Moreover, the IVF rate in the rat medicated serum group was significantly higher than the IVF rate in the blank control and normal rat serum groups and even higher than in the FSH group. These findings suggest that one of effects that YGW uses to reinforce the “Kidney Yang” deficiency is to promote oocyte maturation and subsequent fertilization competence.

However, no significant difference in the GVBD rate was found among the groups in our present study. As mentioned above, although oocyte GVBD is initiated by gonadotrophins, other factors such as epidermal growth factors (EGF) are also involved in the process [18, 19]. The results of our present study may be explained by the fact that HTF was used as the basic culture medium, which contains a number of growth factors such as EGF [20]. Nonetheless, the addition of FSH or rat medicated serum in the HTF medium could induce a significant higher percentage of PB1 oocytes and subsequent IVF rate when compared to the blank control and normal rat serum groups.

Interestingly, the IVF rate in the rat medicated serum group was even higher than the IVF rate in the FSH group, although no significant difference in the percentage of PB1 oocytes was observed between the two groups. This result suggests that the YGW formula not only regulate the process of oocyte maturation but also has more effect on subsequent fertilization competence than FSH.

So what are underlying mechanisms of the effects of YGW on oocyte nuclear maturation? As mentioned earlier, oocytes maturation is initiated by a surge of gonadotrophins. The gonadotrophins bind to their receptors in cumulus granulosa cells resulting in an increased production of cAMP. The elevated cAMP production activates PKA in the cumulus granulosa cells which in turn leads to the activation of CREB and MAPK and causes the MPF complex to become active so that the oocytes can resume meiosis [21]. The other signal pathways, such as the PKC-MAPK pathway and the NO-cGMP-PKG pathway, may also be involved in the regulation of meiotic resumption [21], with the PKC-MAPK pathway positively regulating and the NO-cGMP-PKG pathway negatively regulating oocyte maturation [22–24].

**Figure 6:**
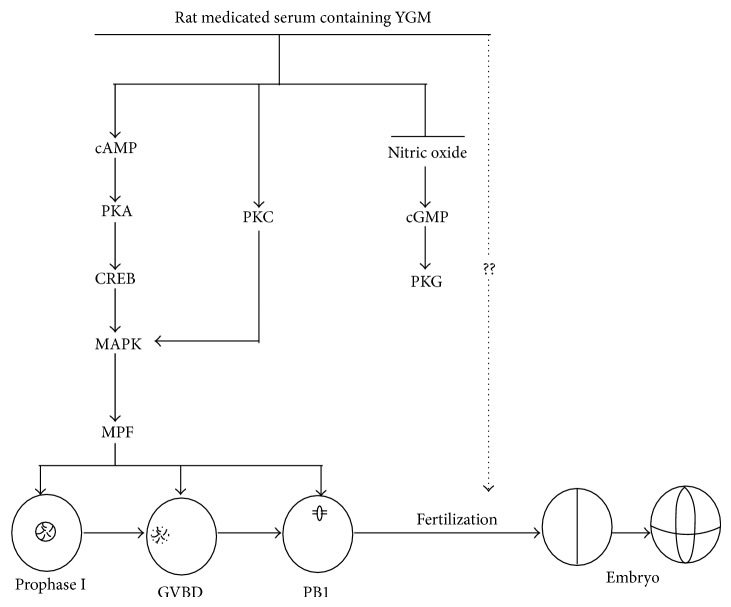
A proposed model for the rat medicated serum containing YGW on oocyte maturation and subsequent fertilization competence. The rat medicated serum induces both the cAMP/PKA/MAPK and PKC/MAPK signal pathways but inhibits the NO/cGMP/PKG signal pathway to enhance oocyte nuclear maturation. Additionally, the rat medicated serum may have better effects on oocyte quality that can increase IVF rate. cAMP: cyclic adenosine 3′5-monophosphate; PKA: protein kinase A; CREB: cAMP response element binding protein; MAPK: mitogen-activated protein kinase; MPF: maturation-promoting factor; PKC: protein kinase C; NO: nitric oxide; cGMP: cyclic guanosine 3′5-monophosphate; PKG: protein kinase G; GVBD: germinal vesicle breakdowns; PB1: first polar body.

In our present study, we showed that both FSH and YGW increased the oocyte cAMP concentration and the expressions of PKA, CREB, MAPK, MPF, and PKC when compared to the blank control and normal rat serum groups. Further, both FSH and YGW decreased the oocyte NO and cGMP concentrations and the expression of PKG when compared to the blank control and normal rat serum groups. Thus, one of the possible underlying mechanisms of YGW may be the regulation of oocyte maturation via signaling pathways including the cAMP/PKA/MAPK pathway, the PKC-MAPK pathway, and the NO-cGMP-PKG pathway, which are similar to those induced by FSH (Figure 6).

The rat medicated serum also showed more beneficial effects than FSH on the IVF rate. It is well known that subsequent oocyte fertilization competence is regulated by a multitude of factors such as cumulus penetration, sperm/oocyte binding, fusion, oocyte activation, sperm processing, and pronuclear (PN) formation [25]. However, the most important factor may be the quality of oocyte maturation, which includes both nuclear and cytoplasmic maturations that are equally essential. Incomplete cytoplasmic maturation may result in fertilization failures although nuclear maturation is normally visualized [26, 27]. A successful fertilization is heavily dependent on the quality of the oocytes, and thus reliant upon the fidelity of oocyte maturation [25, 28, 29]. Therefore, the more beneficial effects of YGW on the IVF rate when compared to the effects of FSH on the IVF rate may be due to the more beneficial effects of YGW on the quality of oocyte maturation. However, no significant difference in oocyte nuclear maturation was found between the YGW and FSH groups. As such, more studies are needed to verify this hypothesis.

The rat medicated serum contains at least three distinct peaks as detected by the HPLC analysis. These biotransformed herbal ingredients may produce more effects on oocyte cytoplasmic maturation and subsequent fertilization competence than FSH. As a result, a higher IVF rate could be obtained in the rat medicated serum group containing the YGW than in the FSH group. However, this hypothesis needs to be experimentally demonstrated in future studies by using isolated herbal ingredients. In addition, future studies are needed to identify the chemical compositions and properties of these herbal integrates.

## 5. Conclusion

In summary, we demonstrated that rat medicated serum containing YGW can enhance the oocyte nuclear maturation* in vitro* via molecular signal pathways similar to those induced by FSH. Further, we also showed that the rat medicated serum can increase the subsequent oocyte fertilization competence after the PB1 stages when compared to FSH treatment. Because a successful fertilization is heavily dependent upon the qualities of the oocytes, and thus reliant on the fidelity of the oocyte maturation and fertilization, our findings should play an important role in the understanding of the pharmacological mechanisms of TCM in treating infertility. This in turn will lead to better clinical practices in the future that combine both TCM and modern reproductive medicine. Nevertheless, further studies are needed to identify which active ingredients and/or biotransformed components are responsible for the underlying mechanisms of the effects of YGW.

## Figures and Tables

**Table 1:** List of the primers used in the qPCR analysis.

Genes	Primers	Amplified size
PKA	TACTTGGCCCCCGAGATTATC GCGAAGAAGGGTGGGTAACC	110 bp

CREB	TGCCCCTGGAGTTGTTATGG CTCTTGCTGCCTCCCTGTTC	110 bp

MAPK	ACCTCAGCAATGACCACATCTG CAGGAGGTTGGAAGGCTTGA	110 bp

MPF	CTCCAGGGAATTGTGTTTTGC AGGCCGAAATCAGCCAGTTT	110 bp

PKC	CGGCTGTACTTCGTCATGGA GAGATCTCGGCTGCGTAGAATAC	110 bp

PKG	TGCGAAGATTCTCATGCTCAA ACTGGCATTTGTGGAGTTTCCT	110 bp

*β*-ACTIN	TGGCATCCATGAAACTACATTCA TGCCTGGGTACATGGTGGTA	110 bp

PKA: protein kinase A; CREB: cAMP-response element binding protein; MAPK: mitogen-activated protein kinase; MPF: maturation-promoting factor; PKC: protein kinase C; PKG: protein kinase G.

**Table 2:** Effects of rat medicated serum containing YGW, FSH, normal rat serum, and culture medium on mouse oocytes maturation *in vitro* and fertilization *in vitro*.

Groups	Number of oocytes	Number of GVBD	Number of PB1	Number of fertilized	Number of cleavage embryos
Control	292	125	91	46	22
Normal serum	277	102	117	60	37
FSH	275	103	118	67	35
Medicated serum	335	133	168	111	59

GVBD: germinal vesicle breakdowns; PB1: first polar body; FSH: follicle-stimulating hormone.
